# A Relay-Signal Model of Nematode Vulval Development

**DOI:** 10.1371/journal.pbio.0020376

**Published:** 2004-09-28

A fundamental question in developmental biology is, how does a multicellular organism develop from a single cell? It's clear that one cell begets two, two beget four, and so on, but how do the newly created cells know which developmental fate to pick? Major insights into this question have come from identifying genes, molecules, and intercellular signaling pathways involved in a wide range of developmental processes. Operating in labyrinthine, often overlapping pathways, intercellular signals determine whether a cell divides, differentiates, migrates, and even lives or dies.

Scientists prefer to work out such problems in organisms with a manageable number of cells for obvious reasons, making the 959-cell soil nematode Caenorhabditis elegans a popular developmental model. C. elegans can exist as either a male or a hermaphrodite, and for some biologists, the hermaphrodite vulva—which consists of just 22 cells—is the perfect system for working out key aspects of intercellular signaling and cell fate.

In a new study, Alex Hajnal and colleagues challenge conventional thinking about vulval cell specification by identifying an enzyme that can amplify a signal's range and help turn three non-vulval precursors into vulval cells. Surprisingly, the enzyme, called ROM-1, accomplishes this feat by acting in the signal-receiving vulval precursor cells, rather than in the signal-sending cell that instructs the vulval cell fates.

The worm vulva forms a bridge between its gonad and the opening to the outer epidermal layer, called the cuticle. In the current model of vulval formation, a group of twelve epidermal cells, called Pn.p cells, lines the ventral surface of the worm. Six of these cells, P3.p–P8.p, the vulval precursor cells (VPCs), have the potential to become vulval cells. During postembryonic development, the anchor cell in the larval gonad secretes an epidermal growth factor (called LIN-3) that activates the EGFR/RAS/MAPK signaling pathway and induces just three of the precursors to differentiate into vulval cells. The VPC closest to the anchor cell, P6.p receives most of the signal, and differentiates into eight vulval cells that form the tube linking the uterus to the gonad. Positioned on either side of P6.p, P5.p and P7.p receive a slightly attenuated signal, which, combined with a lateral signal from P6.p, gives rise to seven vulval cells that form vulval structures. The other three vulval precursors, P3.p, P4.p, and P8.p, it was thought, are too far away to receive the vulval induction signal and fuse into the surrounding epidermis.

**Figure:**
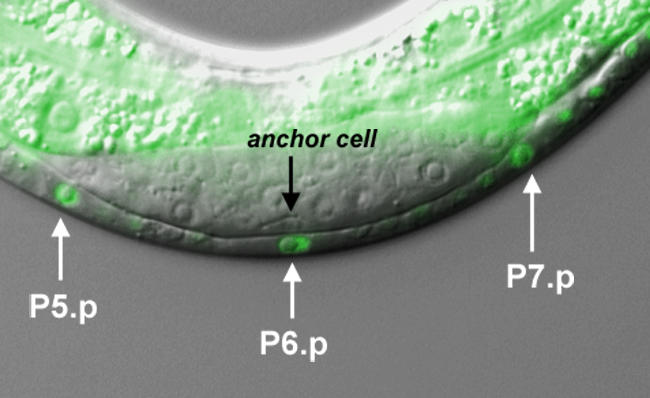
Vulval precursor cells in C. elegans

The LIN-3 epidermal growth factors sit nestled within the cell membrane and must be “processed” to become active, prompting Hajnal and colleagues to look for candidate enzymes that could be doing the processing. They investigated the Rhomboid family of proteases, which are known activators of epidermal growth factor transmembrane proteins, and found one, ROM-1, with the amino acid profile required for catalytic protease activity. After showing that *rom* genes were not required for normal vulval development, the authors had a closer look at their role in vulval cell fate specification. Since loss of ROM-1 reduces the severity of a defect (in this case, multiple vulvas) caused by hyperactivation of the EGFR/RAS/MAPK pathway but has no effect on the precursors closest to the anchor cell, the authors conclude that ROM-1 enhances the EGFR/RAS/MAPK pathway, allowing it to reach the distant P3.p, P4.p, and P8.p precursors.

LIN-3 exists in two variant forms of different lengths, the longer one carrying a stretch of 15 extra amino acids in the region that is cleaved off to yield an active growth factor. Hajnal and colleagues show that ROM-1 only acts on the longer form to regulate the EGFR/RAS/MAPK pathway—and that the ROM-1/LIN-3 interaction occurs in the VPCs, independently of the anchor cell. They go on to propose a two-step model of vulval cell specification in which ROM-1 “extends the range” of the anchor signal, relaying it from the proximal to the more distant precursor cells by promoting the secretion of the long version of LIN-3. In normal development, LIN-3 secretion by the VPCs may serve initially to maintain the differentiation potential of all the precursors, while the anchor cell signal may seal their fates at a later phase.

